# Doctor J. Austin Dunn

**Published:** 1913-05-15

**Authors:** 


### OBITUARY.

Doctor J. Austin Dunn. Dr. Dunn for many years a practitioner of Chicago, formerly of Dayton, Ky., died April 9th, 1913, from Angina Pectoris.

Dr. Dunn was an inventive genius and supplied the profession with several useful appliances. The Dunn Abscess Syringe, Medicine Dropper for Corrosive Agents, Labial Clamps, and Oxyphosphate Cement free from sodium compounds. He was a genial gentleman and had many friends who will learn with regret of his untimely death. He has held many prominent positions in the profession and has always supported the best things professionally.

